# (2-Butoxy­phen­yl)boronic acid

**DOI:** 10.1107/S1600536808000457

**Published:** 2008-01-16

**Authors:** Marek Dąbrowski, Sergiusz Luliński, Janusz Serwatowski

**Affiliations:** a Warsaw University of Technology, Faculty of Chemistry, Noakowskiego 3, 00-664 Warsaw, Poland

## Abstract

The title compound, 2-(CH_3_CH_2_CH_2_CH_2_O)C_6_H_4_B(OH)_2_, exists as a centrosymmetric hydrogen-bonded dimer. Dimers are linked *via* C—H⋯π and π–π [with closest C⋯C contact of 3.540 (3) Å] inter­actions to produce a two-dimensional array.

## Related literature

For related literature, see: Rettig & Trotter (1977 ▶). For the structures of related *ortho*-alkoxy­aryl­boronic acids, see: Dabrowski *et al.* (2006 ▶); Serwatowski *et al.* (2006 ▶); Yang *et al.* (2005 ▶).
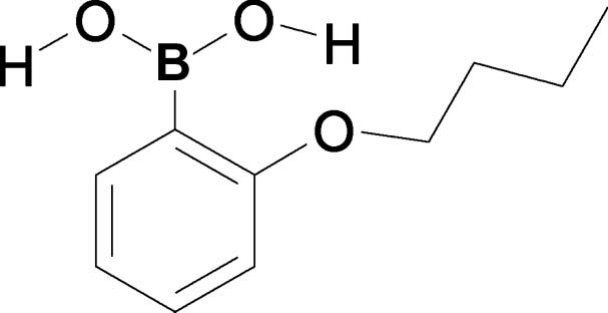


## Experimental

### 

#### Crystal data

C_10_H_15_BO_3_
*M* *_r_* = 194.03
Monoclinic, 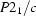

*a* = 7.4809 (4) Å
*b* = 15.3510 (7) Å
*c* = 9.2824 (4) Å
β = 94.299 (4)°
*V* = 1062.98 (9) Å^3^
*Z* = 4
Mo *K*α radiation
μ = 0.09 mm^−1^
*T* = 102 (2) K
0.74 × 0.47 × 0.32 mm

#### Data collection

Kuma KM4 CCD diffractometer
Absorption correction: multi-scan (*CrysAlis RED*; Oxford Diffraction, 2005 ▶) *T*_min_ = 0.92, *T*_max_ = 0.97
9363 measured reflections
2419 independent reflections
1924 reflections with *I* > 2σ(*I*)
*R*_int_ = 0.012

#### Refinement

*R*[*F* ^2^ > 2σ(*F* ^2^)] = 0.032
*wR*(*F* ^2^) = 0.096
*S* = 1.14
2419 reflections
188 parameters
All H-atom parameters refined
Δρ_max_ = 0.35 e Å^−3^
Δρ_min_ = −0.16 e Å^−3^

### 

Data collection: *CrysAlis CCD* (Oxford Diffraction, 2005 ▶); cell refinement: *CrysAlis RED* (Oxford Diffraction, 2005 ▶); data reduction: *CrysAlis RED*; program(s) used to solve structure: *SHELXS97* (Sheldrick, 2008 ▶); program(s) used to refine structure: *SHELXL97* (Sheldrick, 2008 ▶); molecular graphics: *DIAMOND* (Brandenburg, 1999 ▶); software used to prepare material for publication: *SHELXL97*.

## Supplementary Material

Crystal structure: contains datablocks I, global. DOI: 10.1107/S1600536808000457/tk2240sup1.cif

Structure factors: contains datablocks I. DOI: 10.1107/S1600536808000457/tk2240Isup2.hkl

Additional supplementary materials:  crystallographic information; 3D view; checkCIF report

## Figures and Tables

**Table 1:** Hydrogen-bond and C—H⋯π geometry (Å, °)

*D*—H⋯*A*	*D*—H	H⋯*A*	*D*⋯*A*	*D*—H⋯*A*
O2—H2O⋯O1	0.864 (16)	1.900 (16)	2.6547 (9)	145.1 (13)
O3—H3O⋯O2^i^	0.909 (15)	1.870 (15)	2.7776 (9)	175.8 (13)
C7—H7*A*⋯C1^ii^	0.992 (10)	2.939 (10)	3.8346 (12)	150.7 (8)
C7—H7*A*⋯C2^ii^	0.992 (10)	3.103 (10)	4.0850 (12)	170.7 (8)
C7—H7*A*⋯C6^ii^	0.992 (10)	2.965 (10)	3.6436 (13)	126.5 (7)

Symmetry codes: (i) 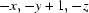; (ii) 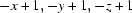.

**Fig. 1.**
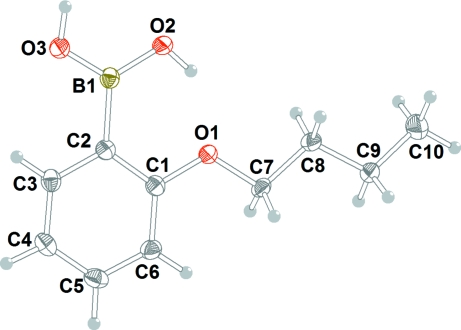
The molecular structure of (I). Displacement ellipsoids are drawn at the 50% probability level.

**Fig. 2.**
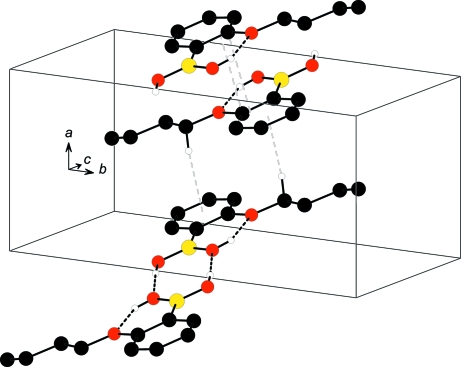
The crystal packing in (I) showing hydrogen-bonding, C—H–π and π–π interactions as dashed lines.

## supplementary crystallographic information

### Comment

The boronic acid group is known to support supramolecular organization due to intermolecular hydrogen bonding. ortho-Substituents in the aryl ring may significantly influence the structural properties of arylboronic acids. There are a few structures of ortho-alkoxyarylboronic acids available in the literature, i.e. those reported by Yang et al. (2005), Serwatowski et al. (2006) and Dabrowski et al. (2006). We were interested in studying the effect of a longer alkoxy chain on the structural characteristics of the related compound, (I).

The molecular structure of (I) shows the entire molecule to be essentially planar, Fig. 1 & Table 1. The mean planes through the boronic and butoxy groups are approximately co-planar with the aromatic ring. The boronic group has an exo–endo conformation. The endo-oriented OH group forms an intramolecular O—H···O bond with the butoxy-O atom, Table 2. As a result, a nearly planar six-membered ring is formed. This motif has been observed in the structures of related ortho-alkoxyarylboronic acids. Monomeric molecules form hydrogen-bonded centrosymmetric dimers typical of boronic acids (Rettig & Trotter, 1977). The crystal packing in (I) features a parallel arrangement of hydrogen-bonded dimers (Fig. 2). It is stabilized in terms of CH-π interactions between the H7a atom of the butoxy group and the aromatic ring of the adjacent molecule: the distance of H7A from the ring centre is 2.777 (11) Å [symmetry code (ii): 1 - x, 1 - y, 1 - z]. As a result, a centrosymmetric dimeric motif can be distinguished. In addition, weak π–π interactions between a pair of aromatic rings lead to their face-to-face center-to-edge stacking with the shortest contact between two C atoms C1—C1^iii^ = 3.540 (3) Å [symmetry code (iii): -x, 1 - y, 1 - z]. The other two π–π interactions are C1···C2^iii^ = 3.594 (5) Å and C1···C6^iii^ = 3.819 (3) Å. Thus, alternate CH-π and π–π interactions result in formation of a two-dimensional array. In conclusion, the hydrogen-bonded dimeric structure of (I) is typical of boronic acids whereas the unique secondary supramolecular assembly is achieved due to weaker CH-π and π–π interactions.

### Experimental

Crystals suitable for the X-ray diffraction analysis were grown by slow evaporation of a solution of the acid (0.2 g) in acetone/water (20 ml, 1:1).

### Refinement

All H atoms were located in difference syntheses and refined freely. The range of O—H distances = 0.864 (16) to 0.909 (15) Å and range of C—H distances = 0.954 (11) to 1.030 (10) Å.

### Crystal data

**Table:**

C_10_H_15_BO_3_	*F*_000_ = 416
*M**_r_* = 194.03	*D*_x_ = 1.212 Mg m^−^^3^
Monoclinic, *P*2_1_/*c*	Mo *K*α radiation λ = 0.71073 Å
Hall symbol: -P 2ybc	Cell parameters from 7109 reflections
*a* = 7.4809 (4) Å	θ = 2.3–30.0º
*b* = 15.3510 (7) Å	µ = 0.09 mm^−^^1^
*c* = 9.2824 (4) Å	*T* = 102 (2) K
β = 94.299 (4)º	Prismatic, colourless
*V* = 1062.98 (9) Å^3^	0.74 × 0.47 × 0.32 mm
*Z* = 4	

### Data collection

**Table:**

Kuma KM4 CCD diffractometer	2419 independent reflections
Radiation source: fine-focus sealed tube	1924 reflections with *I* > 2σ(*I*)
Monochromator: graphite	*R*_int_ = 0.012
Detector resolution: 8.6479 pixels mm^-1^	θ_max_ = 27.5º
*T* = 102(2) K	θ_min_ = 2.7º
ω scans	*h* = −9→9
Absorption correction: multi-scan(CrysAlis RED; Oxford Diffraction, 2005)	*k* = −19→19
*T*_min_ = 0.92, *T*_max_ = 0.97	*l* = −12→11
9363 measured reflections	

### Refinement

**Table:**

Refinement on *F*^2^	Hydrogen site location: inferred from neighbouring sites
Least-squares matrix: full	All H-atom parameters refined
*R*[*F*^2^ > 2σ(*F*^2^)] = 0.032	*w* = 1/[σ^2^(*F*_o_^2^) + (0.0603*P*)^2^] where *P* = (*F*_o_^2^ + 2*F*_c_^2^)/3
*wR*(*F*^2^) = 0.096	(Δ/σ)_max_ < 0.001
*S* = 1.14	Δρ_max_ = 0.35 e Å^−^^3^
2419 reflections	Δρ_min_ = −0.16 e Å^−^^3^
188 parameters	Extinction correction: SHELXL97 (Sheldrick, 2008), Fc^*^=kFc[1+0.001xFc^2^λ^3^/sin(2θ)]^-1/4^
Primary atom site location: structure-invariant direct methods	Extinction coefficient: 0.015 (3)
Secondary atom site location: difference Fourier map	

### Special details

**Table:**

Geometry. All e.s.d.'s (except the e.s.d. in the dihedral angle between two l.s. planes) are estimated using the full covariance matrix. The cell e.s.d.'s are taken into account individually in the estimation of e.s.d.'s in distances, angles and torsion angles; correlations between e.s.d.'s in cell parameters are only used when they are defined by crystal symmetry. An approximate (isotropic) treatment of cell e.s.d.'s is used for estimating e.s.d.'s involving l.s. planes.
Refinement. Refinement of *F*^2^ against ALL reflections. The weighted *R*-factor *wR* and goodness of fit *S* are based on *F*^2^, conventional *R*-factors *R* are based on *F*, with *F* set to zero for negative *F*^2^. The threshold expression of *F*^2^ > σ(*F*^2^) is used only for calculating *R*-factors(gt) *etc*. and is not relevant to the choice of reflections for refinement. *R*-factors based on *F*^2^ are statistically about twice as large as those based on *F*, and *R*- factors based on ALL data will be even larger.

### Fractional atomic coordinates and isotropic or equivalent isotropic displacement parameters (Å^2^)

**Table:**

	*x*	*y*	*z*	*U*_iso_*/*U*_eq_	
C1	0.22865 (11)	0.48929 (6)	0.46605 (10)	0.0187 (2)	
C2	0.15354 (11)	0.42983 (6)	0.36299 (10)	0.0181 (2)	
C3	0.12666 (12)	0.34464 (6)	0.41017 (11)	0.0213 (2)	
C4	0.16761 (12)	0.31891 (6)	0.55234 (11)	0.0242 (2)	
C5	0.23872 (12)	0.37970 (7)	0.65050 (10)	0.0244 (2)	
C6	0.27140 (12)	0.46477 (6)	0.60865 (10)	0.0219 (2)	
C7	0.32860 (12)	0.63751 (6)	0.51594 (10)	0.0196 (2)	
C8	0.34388 (13)	0.72079 (6)	0.43222 (10)	0.0205 (2)	
C9	0.42380 (14)	0.79405 (6)	0.52702 (11)	0.0236 (2)	
C10	0.46998 (16)	0.87395 (7)	0.44072 (13)	0.0324 (3)	
B1	0.09189 (13)	0.45600 (7)	0.20334 (11)	0.0189 (2)	
O1	0.25505 (9)	0.57244 (4)	0.41655 (7)	0.02163 (19)	
O2	0.10984 (9)	0.53922 (4)	0.15252 (7)	0.02530 (19)	
O3	0.01624 (9)	0.39432 (4)	0.11431 (8)	0.0266 (2)	
H2O	0.157 (2)	0.5714 (9)	0.2219 (17)	0.056 (4)*	
H3O	−0.0232 (18)	0.4136 (9)	0.0250 (16)	0.055 (4)*	
H3	0.0761 (15)	0.3037 (7)	0.3414 (12)	0.027 (3)*	
H4	0.1452 (14)	0.2583 (7)	0.5786 (12)	0.028 (3)*	
H5	0.2692 (15)	0.3638 (8)	0.7519 (13)	0.034 (3)*	
H6	0.3221 (14)	0.5074 (7)	0.6758 (12)	0.029 (3)*	
H7A	0.4484 (14)	0.6175 (7)	0.5556 (10)	0.022 (3)*	
H7B	0.2485 (13)	0.6452 (7)	0.5943 (11)	0.019 (2)*	
H8A	0.4243 (13)	0.7100 (6)	0.3536 (11)	0.022 (3)*	
H8B	0.2185 (14)	0.7378 (7)	0.3876 (12)	0.026 (3)*	
H9A	0.5375 (14)	0.7721 (7)	0.5801 (12)	0.027 (3)*	
H9B	0.3361 (16)	0.8101 (7)	0.5990 (12)	0.030 (3)*	
H10A	0.5591 (18)	0.8578 (8)	0.3676 (15)	0.052 (4)*	
H10B	0.3595 (18)	0.8990 (8)	0.3852 (14)	0.045 (3)*	
H10C	0.5225 (16)	0.9206 (8)	0.5042 (13)	0.045 (4)*	

### Atomic displacement parameters (Å^2^)

**Table:**

	*U*^11^	*U*^22^	*U*^33^	*U*^12^	*U*^13^	*U*^23^
C1	0.0164 (4)	0.0187 (5)	0.0210 (5)	0.0035 (3)	0.0007 (3)	0.0005 (4)
C2	0.0152 (4)	0.0192 (5)	0.0200 (4)	0.0025 (3)	0.0009 (3)	0.0001 (4)
C3	0.0193 (5)	0.0195 (5)	0.0251 (5)	0.0011 (4)	0.0010 (4)	−0.0008 (4)
C4	0.0241 (5)	0.0203 (5)	0.0284 (5)	0.0027 (4)	0.0032 (4)	0.0056 (4)
C5	0.0242 (5)	0.0281 (5)	0.0209 (5)	0.0060 (4)	0.0012 (4)	0.0053 (4)
C6	0.0229 (5)	0.0233 (5)	0.0190 (5)	0.0035 (4)	−0.0016 (4)	−0.0015 (4)
C7	0.0212 (5)	0.0204 (5)	0.0167 (4)	0.0003 (3)	−0.0026 (4)	−0.0041 (4)
C8	0.0223 (5)	0.0216 (5)	0.0171 (5)	0.0011 (4)	−0.0020 (4)	−0.0013 (4)
C9	0.0306 (5)	0.0193 (5)	0.0200 (5)	0.0018 (4)	−0.0031 (4)	−0.0021 (4)
C10	0.0397 (6)	0.0234 (5)	0.0332 (6)	−0.0038 (5)	−0.0032 (5)	0.0012 (5)
B1	0.0179 (5)	0.0184 (5)	0.0205 (5)	0.0009 (4)	0.0011 (4)	−0.0009 (4)
O1	0.0289 (4)	0.0173 (4)	0.0176 (3)	−0.0030 (3)	−0.0047 (3)	−0.0005 (3)
O2	0.0369 (4)	0.0203 (4)	0.0176 (4)	−0.0063 (3)	−0.0057 (3)	−0.0002 (3)
O3	0.0363 (4)	0.0194 (4)	0.0225 (4)	−0.0020 (3)	−0.0090 (3)	−0.0008 (3)

### Geometric parameters (Å, °)

**Table:**

C1—O1	1.3758 (11)	C7—H7B	0.983 (10)
C1—C6	1.3902 (13)	C8—C9	1.5223 (13)
C1—C2	1.4083 (13)	C8—H8A	0.994 (10)
C2—C3	1.3984 (13)	C8—H8B	1.030 (10)
C2—B1	1.5710 (13)	C9—C10	1.5189 (14)
C3—C4	1.3895 (13)	C9—H9A	1.009 (11)
C3—H3	0.954 (11)	C9—H9B	1.001 (12)
C4—C5	1.3825 (14)	C10—H10A	1.017 (14)
C4—H4	0.980 (10)	C10—H10B	1.017 (13)
C5—C6	1.3895 (14)	C10—H10C	0.990 (13)
C5—H5	0.982 (12)	B1—O3	1.3526 (12)
C6—H6	0.962 (12)	B1—O2	1.3718 (12)
C7—O1	1.4408 (10)	O2—H2O	0.864 (16)
C7—C8	1.5050 (13)	O3—H3O	0.909 (15)
C7—H7A	0.992 (10)		
			
O1—C1—C6	122.72 (9)	C7—C8—C9	111.76 (7)
O1—C1—C2	115.75 (8)	C7—C8—H8A	108.1 (6)
C6—C1—C2	121.53 (9)	C9—C8—H8A	108.3 (6)
C3—C2—C1	116.92 (8)	C7—C8—H8B	108.7 (6)
C3—C2—B1	119.71 (8)	C9—C8—H8B	110.6 (6)
C1—C2—B1	123.30 (8)	H8A—C8—H8B	109.3 (8)
C4—C3—C2	122.46 (9)	C10—C9—C8	112.75 (8)
C4—C3—H3	119.8 (6)	C10—C9—H9A	108.1 (6)
C2—C3—H3	117.8 (6)	C8—C9—H9A	108.4 (6)
C5—C4—C3	118.72 (9)	C10—C9—H9B	109.8 (6)
C5—C4—H4	122.8 (6)	C8—C9—H9B	108.6 (6)
C3—C4—H4	118.5 (6)	H9A—C9—H9B	109.1 (9)
C4—C5—C6	121.15 (9)	C9—C10—H10A	110.0 (7)
C4—C5—H5	120.9 (7)	C9—C10—H10B	111.4 (7)
C6—C5—H5	118.0 (7)	H10A—C10—H10B	107.6 (11)
C5—C6—C1	119.20 (9)	C9—C10—H10C	111.5 (7)
C5—C6—H6	121.8 (7)	H10A—C10—H10C	108.8 (10)
C1—C6—H6	119.0 (7)	H10B—C10—H10C	107.4 (10)
O1—C7—C8	107.37 (7)	O3—B1—O2	119.28 (8)
O1—C7—H7A	108.3 (6)	O3—B1—C2	118.50 (8)
C8—C7—H7A	110.7 (6)	O2—B1—C2	122.22 (8)
O1—C7—H7B	109.4 (6)	C1—O1—C7	119.14 (7)
C8—C7—H7B	110.7 (6)	B1—O2—H2O	108.7 (10)
H7A—C7—H7B	110.3 (8)	B1—O3—H3O	114.9 (9)
			
O1—C1—C2—C3	179.49 (7)	C2—C1—C6—C5	−0.21 (14)
C6—C1—C2—C3	−1.00 (13)	O1—C7—C8—C9	−179.00 (7)
O1—C1—C2—B1	−3.50 (13)	C7—C8—C9—C10	170.24 (9)
C6—C1—C2—B1	176.01 (8)	C3—C2—B1—O3	−1.04 (13)
C1—C2—C3—C4	1.34 (13)	C1—C2—B1—O3	−177.97 (8)
B1—C2—C3—C4	−175.78 (8)	C3—C2—B1—O2	177.95 (8)
C2—C3—C4—C5	−0.45 (14)	C1—C2—B1—O2	1.02 (14)
C3—C4—C5—C6	−0.84 (14)	C6—C1—O1—C7	−0.53 (12)
C4—C5—C6—C1	1.16 (14)	C2—C1—O1—C7	178.98 (8)
O1—C1—C6—C5	179.27 (8)	C8—C7—O1—C1	179.46 (7)

### Hydrogen-bond geometry (Å, °)

**Table:**

*D*—H···*A*	*D*—H	H···*A*	*D*···*A*	*D*—H···*A*
O2—H2O···O1	0.864 (16)	1.900 (16)	2.6547 (9)	145.1 (13)
O3—H3O···O2^i^	0.909 (15)	1.870 (15)	2.7776 (9)	175.8 (13)
C7—H7A···C1^ii^	0.992 (10)	2.939 (10)	3.8346 (12)	150.7 (8)
C7—H7A···C2^ii^	0.992 (10)	3.103 (10)	4.0850 (12)	170.7 (8)
C7—H7A···C6^ii^	0.992 (10)	2.965 (10)	3.6436 (13)	126.5 (7)

Symmetry codes: (i) −*x*, −*y*+1, −*z*; (ii) −*x*+1, −*y*+1, −*z*+1.
